# Identification of *N*-acyl-l-homoserine lactones produced by non-pigmented *Chromobacterium aquaticum* CC-SEYA-1^T^ and pigmented *Chromobacterium subtsugae* PRAA4-1^T^

**DOI:** 10.1007/s13205-011-0029-1

**Published:** 2011-10-14

**Authors:** P. D. Rekha, Chiu-Chung Young, A. B. Arun

**Affiliations:** 1 Yenepoya Research Center, Yenepoya University, University Road, Deralakatte, Mangalore, 575 018 Karnataka India; 2 Department of Soil and Environmental Sciences, National Chung-Hsing University, Taichung, 402 Taiwan

**Keywords:** *Chromobacterium*, *N*-acyl homoserine lactones, Violacein, Quorum sensing

## Abstract

Many members of the genus *Chromobacterium* produce violacein, a characteristic purple pigment which is induced by small diffusible *N*-acyl homoserine lactones (AHL) quorum-sensing molecules. In this study, the production of AHL of the non-pigmented *C. aquaticum* CC-SEYA-1^T^ and the pigmented *C. subtsugae* PRAA4-1^T^ were determined by using a CV026 biosensor assay. The profile of AHL was identified from the extracts of stationary phase cultures using gas chromatography–mass spectroscopy (GC–MS) and thin layer chromatography (TLC). CV026 biosensor assay revealed that both the non-pigmented *C. aquaticum* CC-SEYA-1^T^ and the pigmented *C. subtsugae* PRAA4-1^T^ produced AHL molecules, which were identified, respectively, as *N*-octanoyl homoserine lactone (OHL) [also known as C-8 homoserine lactone (C8-HSL)] and *N*-hexanoyl homoserine lactone (HHL) [also known as C-6 homoserine lactone (C6-HSL)]. The pigment produced by *C. subtsugae* PRAA4-1^T^ was similar to that of *Chromobacterium violaceum* ATCC12472^T^ but no characteristic visible spectral peaks of the pigment were observed in the extracts of *C. aquaticum* CC-SEYA-1^T^. In addition, *C. aquaticum* CC-SEYA-1^T^ and *C. subtsugae* PRAA4-1^T^ showed hemolytic activities.

## Introduction

Quorum sensing (QS) is an important process of cell to cell communication in which cells produce, detect, and respond to extracellular signal molecules called autoinducers such as *N*-acyl-homoserine lactone (AHL) (Fuqua and Greenberg 2002). Quorum sensing regulates different types of physiological functions, such as biofilm formation, bioluminescence, DNA exchange etc., in diverse species of Gram negative bacteria (Williams et al. 2007; Whitehead et al. 2001) and many pathogenic bacteria use QS system to control genes required for expression of virulence (Ng and Bassler 2009).

Members of the genus *Chromobacterium* have been isolated from both the aquatic and terrestrial environments. They possess a versatile energy metabolism which enables them to live in diverse environmental conditions. Of the six species described, four are pigmented, whereas the remaining two are non-pigmented (Table 1). Of these, *Chromobacteriumviolaceum* is the most studied member of the genus. It often infects humans and causes bacteremia (Vijayan et al. 2009; Chattopadhyay et al. 2002). Its deep violet pigment, violacein possesses broad-spectrum antibacterial activities (Duran et al. 1994). Although the physiological function of this pigment is unclear, the mechanism of control of violacein production is well understood (Chen et al. 2011; Antonio and Creczynski-Pasa 2004; McClean et al. 1997). Studies have shown that violacein is arranged in an operon consisting of *vio*D, *vio*C, *vio*B, and *vio*A genes which is regulated through a quorum-sensing system mediated by AHL (August et al. 2000).

**Table 1:** Details of the pigmentation and important characteristics of the six described species of the genus *Chromobacterium*

No.	Species name	Pigment	Important activity	References
1	*C. violaceum* ATCC 12472^T^	Violet pigment	Violacein pigment, QS, antimicrobial, anticancer, pathogenic, hemolytic, produce HHL	Sebek and Jager (1962); de Boisbaudran (1882); Duran et al. (1994); McClean et al. (1997); Vijayan et al. (2009); Chattopadhyay et al. (2002)
2	*C. subtsugae* PRAA4-1^T^	Violet pigment	Insecticidal against Colarado potato beetle, hemolytic (this study)	Martin et al. (2007a, b)
3	*C. aquaticum* CC-SEYA-1^T^	No pigment	Hemolytic (this study)	Young et al. (2008)
4	*C. haemolyticum* MDA0585^T^	No pigment	Hemolytic, higher resistance to antibiotics	Han et al. (2008)
5	*C. piscinae* LMG 3947^T^	Violet pigment	–	Kämpfer et al. (2009)
6	*C. psuedoviolaceum* LMG 3953^T^	Violet pigment	–	Kämpfer et al. (2009)

Other studies have shown that violacein provides a protective mechanism by which the bacteria avoid predation (Matz et al. 2004). Violacein also has commercial uses in medicine due to its broad-spectrum bactericidal and antitumor activities (Duran and Menk 2001). Studies of the genome sequence of *C. violaceum* have revealed ORFs that encode products of biotechnological and medical interest (Brazilian Genome Project Consortium 2003), as a consequence, an additional five species have been described recently but little to no details on the biology of these isolates is known.

We report here results on our studies of AHL in, and hemolytic potential of, *Chromobacterium aquaticum* CC-SEYA-1^T^ (Young et al. 2008) and *Chromobacterium subtsugae* PRAA4-1^T^ (Martin et al. 2007a) and the comparison of violacein produced by *C. subtsugae* PRAA4-1^T^ compared to the violacein of *C. violaceum* ATCC 12472^T^.

## Materials and methods

### Bacterial strains and culture conditions

*C. aquaticum* CC-SEYA-1^T^ and *C. subtsugae* PRAA4-1^T^ were kindly provided by P.A. Martin (Insect Biocontrol Laboratory, US Department of Agriculture, Agriculture Research Service, 10300 Baltimore Ave, Beltsville, MD, USA), *C. violaceum* ATCC 12472^T^ was purchased from Food Industry Research and Development Institute, Bioresource Collection and Research Center (FIRDI, BCRC), Taiwan and *C. violaceum* CV026, a mini-Tn5 mutant of *C. violaceum* ATCC 31532, which was used as the biosensor strain, was a gift from Prof. Dr. Hsin-Chih Lai (Department of Medical Biotechnology and Laboratory Sciences, Chang Gang University, Taiwan).

Unless indicated otherwise, all cultures were grown with shaking (140 rpm) in Luria Bertani (LB) broth at 30 °C and absorbency recorded at 585 and 660 nm. For detection of hemolytic activity, *C. violaceum* ATCC 12472^T^, *C. aquaticum* CC-SEYA-1^T^, and *C. subtsugae* PRAA4-1^T^ were grown on sheep blood agar media (TPM, Taiwan) and incubated at 24–48 h at 30 °C. Formation of clear zones around the colonies indicated hemolytic activity.

### AHL standards

AHL in the following forms were purchased from Fluka (Buchs, Switzerland): *N*-butanoyl (BHL), *N*-hexanoyl (HHL) and *N*-octanoyl (OHL), *N*-decanoyl (DHL), *N*-dodecanoyl (dDHL) and *N*-tetradecanoyl (tDHL). Methanol was purchased from Sigma-Aldrich (Buchs, Switzerland). For GC/MS analyses stock solutions of AHL (1 mg ml^−1^ in methanol) were diluted with methanol to final concentration of 100 μg ml^−1^. For the biosensor assay, HHL and OHL were diluted to 40 and 45.5 ng ml^−1^ concentration, respectively, in ethyl acetate.

### Detection of AHL by agar plate assay

Screening for AHL production was performed by using the agar plate biosensor bioassay with *C. violaceum* CV026 as the reporter strain (Ravn et al. 2001). Briefly, *C. aquaticum* CC-SEYA-1^T^ or *C. subtsugae* PRAA4-1^T^ were streaked parallel to the streaked *C. violaceum* CV026 strain on LB agar plates and incubated at 30 °C for 48 h.

### Isolation, purification, and characterization of AHL from cell-free supernatants

Culture supernatants (100 ml) of *C. aquaticum* CC-SEYA-1^T^ and *C. subtsugae* PRAA4-1^T^, that had been grown in LB media to stationary phase, were extracted three times with chloroform (1:1 supernatant/chloroform), and the organic phase was pooled and passed over a column filled with anhydrous magnesium sulfate. The samples containing AHL were evaporated to dryness under a thin stream of nitrogen gas at room temperature. The dried residue was dissolved in 5 ml methanol, transferred to small glass vials and concentrated further to 1 ml before analysis by GC/MS.

### GC/MS and AHL standards

A GC system (Trace GC Ultra, Thermo, USA) interfaced to a single quadrupole mass selective detector DSQII (Thermo, USA) and controlled by the software Xcaliber^™^ (Version 1.4 SR1, Thermo electron Co. Inc. USA) equipped with an auto-sampler (AS 3000, Thermo, USA) and a DB-5MS MSD capillary column, 30 m × 0.25 mm ID, 0.25 μm was used for AHL characterization. Helium (99.999%) at the flow rate of 0.8 ml min^−1^ was used as the carrier gas. The GC oven temperature was increased from 150 (3 min hold) to 275 °C at a rate of 15 °C min^−1^. The injector was kept at 200 °C, the transfer line at 280 °C, and the ion source at 230 °C. The ionization energy was 70 eV and the mass spectrometer was run at SIM (single ion monitoring) mode at *m/z* 143 (Cataldi et al. 2007). AHL molecules were identified and quantified by comparing the retention times and peak areas against AHL standards. For this, standard stock solutions of AHL (1 mg ml^−1^ in methanol) were diluted with methanol to final concentration of 100 μg ml^−1^. Five microliters of standard or the test sample was injected using the auto-sampler in split mode (150:1) under the specified conditions.

### Thin layer chromatography (TLC)

Thin layer chromatography was performed on C18 reversed-phase plates using a methanol/water (60:40 v/v) solvent system essentially as described by Shaw et al. (1997) but with *C. violaceum* CV026 used instead of *A. tumefaciens* mutant. Twenty microliters of synthetic AHL solutions in acetonitrile (100 μg ml^−1^) or extracts of culture supernatants (10 ml extracted with equal volume of chloroform, concentrated, and re-dissolved in 1 ml of acetonitrile) were spotted onto aluminum backed RP18 TLC plates (Merck, Germany). The presence of AHL was detected after drying the TLC plates by overlaying a thin film of 0.3% (w/v) LB agar seeded with *C. violaceum* CV026 (10^8^ CFU). After overnight incubation at 30 °C, AHLs were located as purple spots on a white background.

### Quantification of AHL using biosensor broth assay

The quantification of the AHL present in the two species was determined by using biosensor strain *C. violaceum* CV026 as described by Blosser and Gray (2000). For this, spent supernatant (10 ml) from stationary phase cultures of *C. aquaticum* CC-SEYA-1 and *C. subtsugae* PRAA4-1^T^ were extracted with ethyl acetate thrice. The extracts were concentrated to 1 ml served as exogenous autoinducers and added to 10 ml LB broth inoculated with *C. violaceum* CV026 for violacein production. 40 ng of synthetic (Fluka, Buchs, Switzerland) HHL and 45.5 ng OHL were dissolved in 1 ml ethyl acetate and were added to 10 ml LB broth inoculated with *C. violaceum* CV026. Response of biosensor cells to the addition of spent medium extract of the two test bacteria or synthetic AHL was calculated as the ratio of absorbance of butanol extract containing violacein to the bioassay culture density and the values were referred to as violacein units.

## Results and discussions

**Fig. 1:**
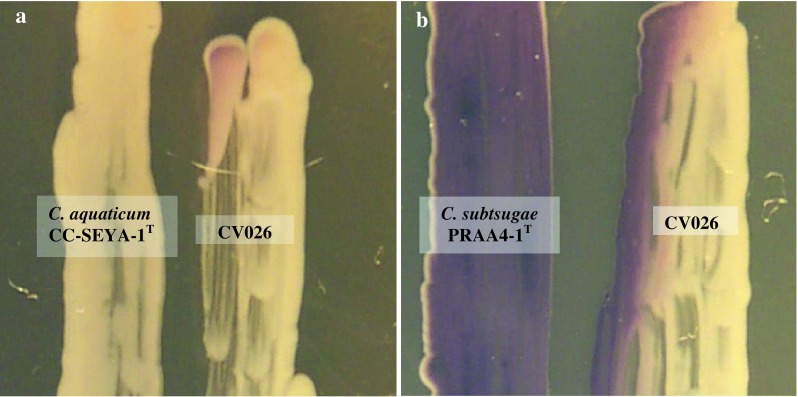
Biosensor plate bioassay showing the induction of violacein in *C. violaceum* CV026 biosensor strain by stationary phase cell-free supernatants of **a***C. aquaticum* CC-SEYA-1^T^ and **b***C. subtsugae* PRAA4-1^T^

**Fig. 2:**
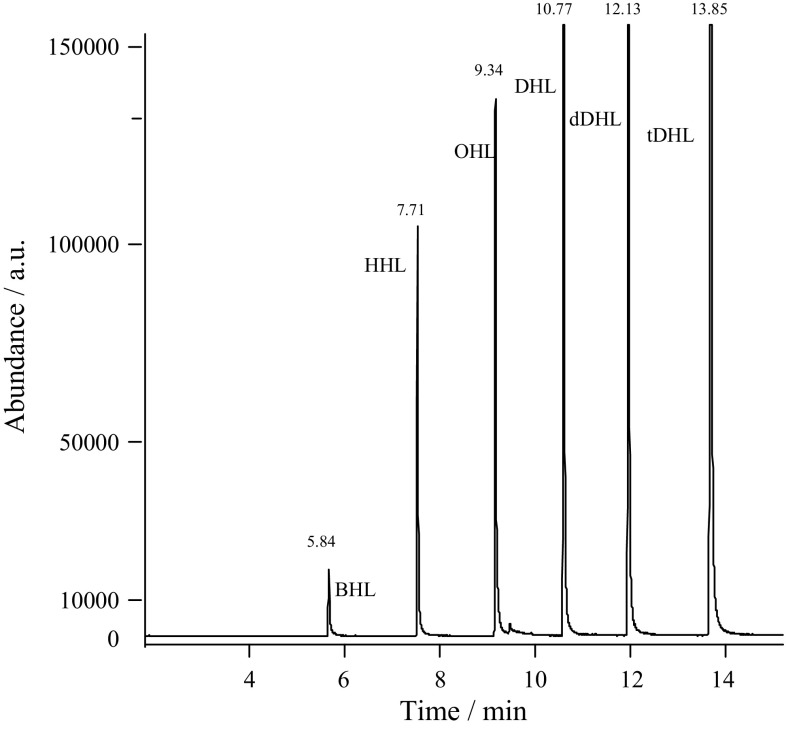
GC–MS chromatogram in SIM mode at *m/z* 143 of standard AHL compounds with their retention times. Synthetic AHLs used are, *N*-butanoyl (BHL), *N*-hexanoyl (HHL), *N*-octanoyl (OHL), *N*-decanoyl (DHL), *N*-dodecanoyl (dDHL), and *N*-tetradecanoyl (tDHL) homoserine lactones

**Fig. 3:**
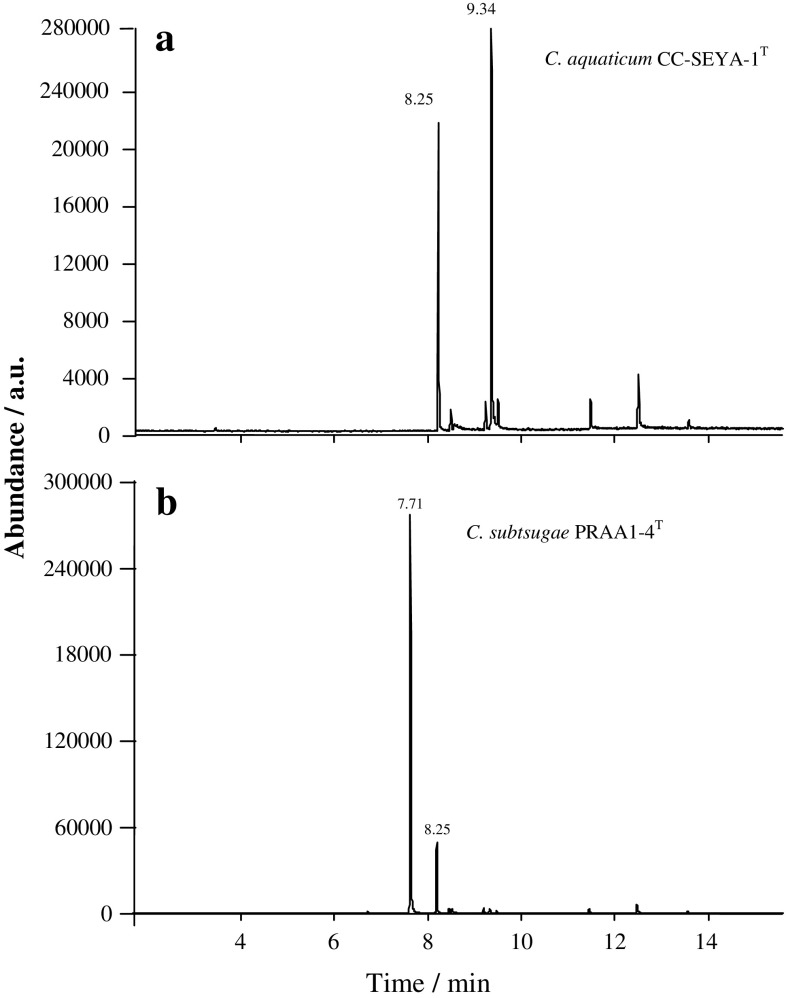
GC–MS chromatogram in SIM mode at *m/z* 143 of AHL compounds from cell-free supernatants extracted in organic solvents of **a***C. aquaticum* CC-SEYA-1^T^ and **b***C. subtsugae* PRAA4-1^T^ with their retention times

Production of AHL was detected using a biosensor plate assay with *C*. *violaceum* CV026. In this assay, violacein is produced by the mutant strain *C*. *violaceum* CV026 only in the presence of exogenous AHL (McClean et al. 1997). Induction of violacein in *C. violaceum* CV026 by *C. aquaticum* CC-SEYA-1^T^ was weak compared to that of *C. subtsugae* PRAA4-1^T^ (Fig. 1). Based on these positive results, the identification of AHL compounds was carried out using GC/MS. The AHL standards showed clearly resolved peaks at different retention times (Fig. 2). The culture supernatants of *C. aquaticum* CC-SEYA-1^T^ produced a predominant peak with retention time of 9.34 min (Fig. 3a) which corresponds to OHL. For the pigmented isolate, *C. subtsugae* PRAA4-1T, a major peak with a retention time of 7.71 min was observed (Fig. 3b) and was identified as HHL. The minor peak at 8.25 min was observed for both *C.subtsugae* PRAA4-1^T^ and *C. aquaticum* CC-SEYA-1^T^, but did not correspond to any of the standards used. From the TLC, it was further confirmed that both the species studied produced only AHL compound each (data not given) and hence the peak at 8.25 min in the GC/MS chromatogram did not correspond to any AHL molecule.

Among the studied species of *Chromobacterium*, OHL was present only in the non-pigmented *C. aquaticum* CC-SEYA-1^T^ as the major AHL and the induction of violacein by the OHL present in the *C. aquaticum* spent cultures was weak as observed in the biosensor broth assay (Table 2). It was also observed that violacein production in CV026 by synthetic OHL was less compared to the HHL even when equal amounts of synthetic compounds were used in the assay. This is consistent with the idea that HHL is the cognate autoinducer for violacein production in *C. violaceum*. Recent studies have shown that there is variation in the dominant type of AHL compounds produced by different *C. violaceum* strains. For example, in *C. violaceum* ATCC 31532, HHL mediates quorum sensing and pigment (violacein) production (McClean et al. 1997), whereas in *C. violaceum* ATCC 12472, several AHLs are present and violacein production is controlled by *N*-(3-hydroxydecanoyl)-l-homoserine lactone (Morohoshi et al. 2008). This would be similar to reports on the presence of many distinguishable AHL compounds with probable autoinducer activities in other Gram negative bacteria (Bruhn et al. 2004).

**Table 2:** Violacein induction in *C. violaceum* CV026 biosensor strain by cell-free supernatants of *C. aquaticum* CC-SEYA-1^T^ and *C. subtsugae* PRAA4-1^T^ and synthetic HHL and OHL

Species name	Cell density (OD_660 nm_)	Violacein (OD_585 nm_)	Violacein units^a^
*C. aquaticum* CC-SEYA-1^T^	0.640 ± 0.01	0.00 ± 0.00	12.16 ± 1.20^1^
*C. subtsugae* PRAA4-1^T^	0.656 ± 0.02	0.17 ± 0.02	181.54 ± 2.84^1^
*C. violaceum* CV026	0.632 ± 0.05	0.00 ± 0.00	130.00 ± 0.68^2^
(Control)			96.22 ± 0.14^3^

^a^Violacein unit is the amount of pigment produced by *C. violaceum* CV026 biosensor strain in response to ^1^autoinducer(s) present in cell-free supernatants of *C. aquaticum* CC-SEYA-1^T^, *C. subtsugae PRAA4*-*1*^*T*^. 10 ml cell-free supernatant from stationary phase cultures were extracted with ethyl acetate and concentrated before use. Synthetic ^2^HHL (4 ng ml^−1^) and ^3^OHL (4.5 ng ml^−1^) in ethyl acetate were used as controls with *C. violaceum* CV026 biosensor strain

In *C. violaceum*, the proteins that synthesize QS-controlled systems are evolutionarily well conserved comprising two adjacent genes *cviI and cviR* homologous to *LuxI and LuxR*, respectively. The AHL mediated QS in *C. violaceum* is believed to have several roles in addition to pigment, hydrogen cyanide, antibiotics, and exoprotease production (Stauff and Bassler 2011; Duran and Menk 2001). Chitinase, elastase, and antibiotic phenazine production are also under the control of QS (Stover et al. 2000; Chernin et al. 1998). In addition, genes regulated by quorum sensing dictate a diversity of physiological traits in various bacteria that are often involved in the establishment of mutualistic symbioses and pathogenic relationships (Visick et al. 2000). It has been reported that QS in *C. violaceum* 31532 is antagonized by long chain AHLs produced by other bacterial species including *C. violaceum* 12472 (Chen et al. 2011). These strategies may be required for effective competition while living in complex environments by controlling the regulation of specific genes or to control specific functions. To establish the role of AHL in the non-pigmented isolates, more specific studies on the QS-controlled gene regulation are necessary. The concentration of violacein produced by *C. subtsugae* PRAA4-1^T^ in comparison with *C. violaceum* ATCC 12472 is presented in Table 2. It is assumed that the production of biologically active compounds is sometimes correlated with the presence of pigments in environmental isolates (Egan et al. 2002). *Chromobacterium* is widely distributed in natural environments where competition and predation are high. For this, defense strategies such as production of pigments and antibiotics controlled by quorum sensing have been evolved. Violacein produced by some of the aquatic biofilm forming bacteria also serves as an important chemical defense against eukaryotic predation (Matz et al. 2008).

**Fig. 4:**
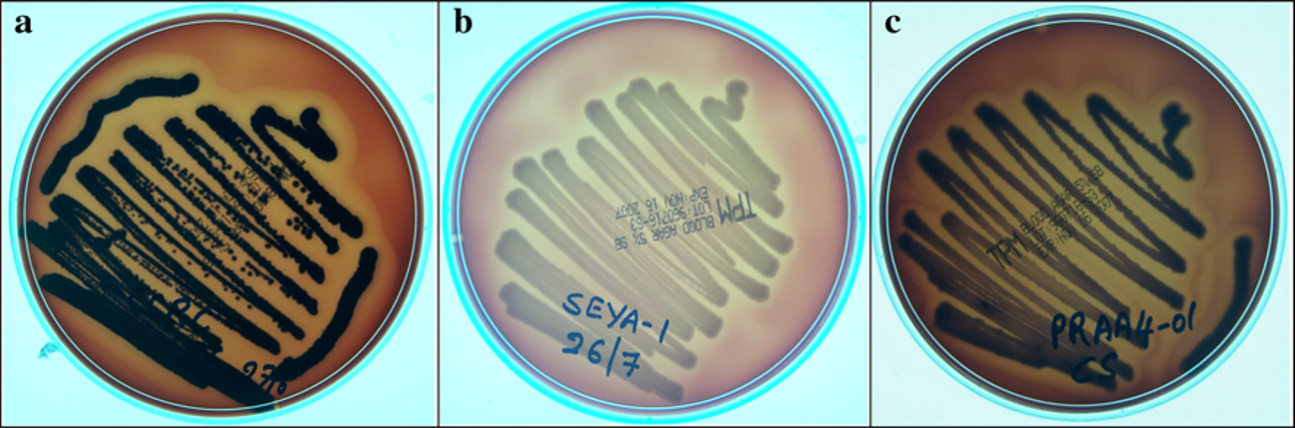
Zones of clearance around colonies of **a***C. violaceum* ATCC 12472, **b***C. aquaticum* CC-SEYA-1^T^, and **c***C. subtsugae* PRAA4-1^T^ on sheep blood agar indicating hemolysis

We observed hemolytic activities in *C. subtsugae* PRAA4-1^T^, *C. aquaticum* CC-SEYA-1^T^, and *C. violaceum* ATCC 12472, (Fig. 4). The pathogenecity of *Chromobacterium* is well established and whole genome studies on *C. violaceum* have identified genes responsible for potential virulence factors including cytolytic toxins (hemolysins), metallo-proteases and lipases (de Britto et al. 2004). A *Serratia* type-hemolysin has also been reported (Brumbach et al. 2007). *C. subtsugae* produces toxins effective against the sweet potato white fly, *Bemisia tabaci* and hence has potential application as biocontrol agent (Martin et al. 2007b). However, it has been proven that, hemolytic activity is not controlled by the AHL mediated QS in *Chromobacterium*. Understanding the pathogenic traits such as hemolytic activity is important to establish biosafety guidelines for the new members of the genus and will open up more clues into the pathogenic nature of these bacteria. A few studies have reported that non-pigmented strains of *Chromobacterium* could also have pathogenic potential comparable to that of the pigmented counterparts (Sivendra and Tan 1977).

From the study on biogeography of violet pigmented *Chromobacterium* species in the Brazilian tropical and subtropical soil water samples, it was revealed that microenvironment barriers such as water and soil play an important role in periodic selection and diversification of these species (Lima-Bittencourt et al. 2010).

*C. violaceum* has attracted a lot of focus due to the presence of QS system and the recent introduction of members to the genus. Many further studies can be taken up exploiting the complex phenomenon related to their biochemical, physiological and their biotechnological uses. More studies should focus on the distribution of AHL molecules for understanding the quorum-sensing phenomenon, and versatility of these bacteria to increase their biotechnological potential.

## References

[CR1] Antonio RV, Creczynski-Pasa TB (2004). Genetic analyses of violacein bio-synthesis by *Chromobacterium violaceum*. Genet Mol Res.

[CR2] August PR, Grossman TH, Minor C (2000). Sequence analyses and functional characterization of the violacein biosynthetic pathway from *Chromobacterium violaceum*. J Mol Microbiol Biotechnol.

[CR3] Blosser RS, Gray KM (2000). Extraction of violacein from *Chromobacterium violaceum* provides a new quantitative bioassay for *N*-acyl homoserine lactone autoinducers. J Microbiol Methods.

[CR4] Brazilian National Genome Project Consortium (2003). The complete genome sequence of *Chromobacterium violaceum* reveals remarkable exploitable bacterial adaptability. Proc Natl Acad Sci USA.

[CR5] Bruhn JB, Christensen AB, Flodgaard LR, Nielsen KF, Larsen TO, Givskov M, Gram L (2004). Presence of acylated homoserine lactones (AHLs) and AHL-producing bacteria in meat and potential role of AHL in spoilage of meat. Appl Environ Microbiol.

[CR6] Brumbach KC, Eason BD, Anderson LK (2007). The *Serratia*-type hemolysin of *Chromobacterium violaceum*. FEMS Microbiol Lett.

[CR7] Cataldi TRI, Bianco G, Palazzo L, Quaranta V (2007). Occurrence of *N*-acyl-l-homoserine lactones in extracts of some Gram-negative bacteria evaluated by gas chromatography–mass spectrometry. Anal Biochem.

[CR8] Chattopadhyay A, Kumar V, Bhat N, Rao P (2002). *Chromobacterium violaceum* infection: a rare but frequently fatal disease. J Pediatr Surg.

[CR9] Chen G, Swem LR, Swem LD (2011). A strategy for antagonizing quorum sensing. Mol Cell.

[CR10] Chernin LS, Winson MK, Thompson JM (1998). Chitinolytic activity in *Chromobacterium violaceum*: substrate analysis and regulation by quorum sensing. J Bacteriol.

[CR11] de Boisbaudran L (1882) Matière colorante se formant dans la colle de farine. CR Acad Sci Paris 9:562–563

[CR12] de Britto CFA, Carvalho CMB, Santos FR (2004). *Chromobacterium violaceum* genome: molecular mechanisms associated with pathogenicity. Genet Mol Res.

[CR13] Duran N, Menk CFM (2001). *Chromobacterium violaceum*: a review of pharmacological and industrial perspectives. Crit Rev Microbiol.

[CR14] Duran N, Antonio RV, Huan M, Pilli RA (1994). Biosynthesis of a trypanocide by *Chromobacterium violaceum*. World J Microbiol Biotechnol.

[CR15] Egan S, James S, Holmström C, Staffan K (2002). Correlation between pigmentation and antifouling compounds produced by *Pseudoalteromonas tunicata*. Environ Microbiol.

[CR16] Fuqua C, Greenberg EP (2002). Listening in on bacteria: acyl-homoserine lactone signaling. Nat Rev Mol Cell Biol.

[CR17] Han XY, Han FS, Segal J (2008). *Chromobacterium haemolyticum* sp. nov., a strongly haemolytic species. Int J Syst Evol Microbiol.

[CR18] Kämpfer P, Busse H-J, Scholz HC (2009). *Chromobacterium piscinae* sp. nov. and *Chromobacterium pseudoviolaceum* sp. nov., from environmental samples. Int J Syst Evol Microbiol.

[CR19] Lima-Bittencourt CI, Costa PS, Hollatz CC, Raposeiras R, Santos FR, Chartone-Souza E, Nascimento AMA (2010). Comparative biogeography of *Chromobacterium* from the neotropics. Antonie Van Leeuwenhoek.

[CR20] Martin PA, Gundersen-Rindal D, Blackburn MB, Buyer J (2007). *Chromobacterium subtsugae* sp. nov., a betaproteobacterium toxic to Colorado potato beetle and other insect pests. Int J Syst Evol Microbiol.

[CR21] Martin PA, Horise E, Aldrich JR (2007). Toxicity of *Chromobacterium subtsugae* to southern green stink bug (Heteroptera: Pentatomidae) and corn rootworm (Coleoptera: Chrysomelidae). J Econ Entomol.

[CR22] Matz C, Deines P, Boenigk J, Arndt H, Eberl L, Kjelleberg S, Jurgens K (2004). Impact of violacein-producing bacteria on survival and feeding of bacterivorous nanoflagellates. Appl Environ Microbiol.

[CR23] Matz C, Webb J, Schupp PJ, Phang SY, Penesyan A, Egan S, Steinberg P, Kjelleberg S (2008). Marine biofilm bacteria evade eukaryotic predation by targeted chemical defense. PLoS One.

[CR24] McClean KH, Winson MK, Fish L, Taylor A, Chhabra SR, Camara M, Daykin M, Lamb JH, Swift S, Bycroft BW, Stewart GS, Williams P (1997). Quorum sensing and *Chromobacterium violaceum*: exploitation of violacein production and inhibition for the detection of *N*-acyl homoserine lactones. Microbiology.

[CR25] Morohoshi T, Kato M, Fukamachi K, Kato N, Ikeda T (2008). *N*-acyl homoserine lactone regulates violacein production in *Chromobacterium violaceum* type strain ATCC 12472. FEMS Microbiol Lett.

[CR26] Ng WL, Bassler BL (2009). Bacterial quorum-sensing network architectures. Annu Rev Genet.

[CR27] Ravn L, Christensen AB, Molin S, Givskov M, Gram L (2001). Methods for detecting acylated homoserine lactones produced by Gram-negative bacteria and their application in studies of AHL-production kinetics. J Microbiol Methods.

[CR28] Sebek OK, Jager H (1962). Divergent pathways of indole metabolism in *Chromobacterium violaceum*. Nature.

[CR29] Shaw PD, Ping G, Daly S, Cha C, Cranan JE, Rinehart KL (1997). Detecting and characterizing acyl-homoserine lactone signal molecules by thin layer chromatography. Proc Natl Acad Sci USA.

[CR30] Sivendra R, Tan SH (1977). Pathogenicity of non-pigmented cultures of *Chromobacterium violaceum*. J Clin Microbiol.

[CR31] Stauff DL, Bassler BH (2011). Quorum sensing in *Chromobacterium violaceum*: DNA recognition and gene regulation by the CviR Receptor. J Bacteriol.

[CR32] Stover CK, Pham XQ, Erwin AL (2000). Complete genome sequence of *Pseudomonas aeruginosa* PAO1, an opportunistic pathogen. Nature.

[CR33] Vijayan AP, Anand MR, Remesh P (2009). *Chromobacterium violaceum* sepsis in an infant. Indian Pediatr.

[CR34] Visick KL, Foster J, Doino J, McFall-Ngai M, Ruby EG (2000). *Vibrio fischeri lux* genes play an important role in colonization and development of the host light organ. J Bacteriol.

[CR35] Whitehead NA, Barnard AML, Slater H, Simpson NJL, Salmond GPC (2001). Quorum-sensing in Gram-negative bacteria. FEMS Microbiol Rev.

[CR36] Williams P, Winzer K, Chan W, Camara M (2007). Look who’s talking: communication and quorum sensing in the bacterial world. Philos Trans R Soc Lond B Biol Sci.

[CR37] Young CC, Arun AB, Lai W-A, Chen WM, Chou JH, Shen FT, Rekha PD, Kempfer P (2008). *Chromobacterium aquaticum* sp. nov., isolated from spring water samples. Int J Syst Evol Microbiol.

